# Implementing PROMs in routine clinical care: a qualitative exploration of GP perspectives

**DOI:** 10.3399/bjgpopen20X101135

**Published:** 2021-01-06

**Authors:** Ian Litchfield, Sheila Greenfield, Grace M Turner, Samuel Finnikin, Melanie J Calvert

**Affiliations:** 1 Institute of Applied Health Research, University of Birmingham College of Medical and Dental Sciences, Birmingham, UK; 2 Centre for Patient Reported Outcomes Research, University of Birmingham, Birmingham, UK; 3 NIHR Birmingham Biomedical Research Centre, University of Birmingham, Birmingham, UK

**Keywords:** qualitative research, family practice, general practitioners, patient perspectives

## Abstract

**Background:**

The recently announced long-term plan for the NHS is based on a model of person-centred care, which relies on the sustained engagement of patients, shared decision making, and capability for self-management. For a primary care service under increasing pressure from an ageing and chronically ill population, Patient Reported Outcome Measures (PROMs) appear capable of supporting many of the requirements for person-centred care, yet little is known of the circumstances of their current implementation or how their use might be optimised.

**Aim:**

To begin the conversation about how successfully PROMs have been integrated into primary care and how their use might be supported.

**Design & setting:**

A qualitative investigation of the perspectives of GPs exploring the use of PROMs as part of routine clinical care in England.

**Method:**

Semi-structured telephone interviews were conducted with GPs from across England. The data were analysed using the Consolidated Framework for Implementation Research (CFIR).

**Results:**

A total of 25 interviews were conducted and GPs described the potential benefit of PROMs in a range of circumstances, but also voiced concerns about their reliability and their potential to constrain consultations. Their flexibility meant they had the potential to be incorporated into existing care processes but only with the requisite logistical support.

**Conclusion:**

Areas that need to be addressed include the creation of a compelling body of evidence of the benefit of PROMs, appropriate training for staff and patients, and a coherent implementation strategy from policymakers and funding bodies.

## How this fits in

Central to the 10-year plan for the future of the NHS is the model of person-centred care, which is reliant on improving patient engagement and ensuring their preferences are assimilated into mutually agreed treatment decisions. One set of tools well placed to meet these requirements and already widely used in general practice are PROMs, yet their introduction has been ad hoc, and surprisingly little is known of their value or the factors influencing their utilisation. The perspectives of GPs are presented in this study as part of the first structured description of the current and potential use of PROMs in UK primary care. It includes the factors influencing their implementation and how they can underpin patient-centred care now and in the future.

## Introduction

In 2019 NHS England launched their long-term plan for the future of health care.^1^ Designed to meet the increasing complexity of people’s needs and expectations, it is considered particularly important to primary care where it is hoped it can ease the mounting pressure on clinicians and help secure the future of the service.^2^ At its centre is a model of person-centred care, which recognises that the priorities, preferences, and capabilities of individual patients must be understood and utilised.^3^ For this model to succeed, ways must be found to improve and sustain its various components, which include patient engagement, health literacy, communication with clinicians, and the overall capability to self-manage.^4^ One set of tools that appear capable of supporting these multiple requirements are PROMs,^5,6^ which are questionnaires designed to be completed by patients to assess their perceptions about the effects of disease and treatment on symptoms, functioning, and quality of life.^6–8^

Originally used for research or as a means of audit or benchmarking,^6,9,10^ there has been a recent shift both internationally^11,12^ and in the UK^9,10^ toward the incorporation of PROMs into care at an individual patient level.^13,14^ Evidence is emerging, predominantly in secondary and ambulatory settings, that using PROMs can lead to improved levels of shared decision making and the delivery of care more responsive to individual needs.^6,8,14,15^ The apparent efficacy and versatility of PROMs has seen them introduced in increasing numbers to primary care for a variety of reasons, which include facilitating discussions between patients and clinicians, and assessing the severity of symptoms (see Table 1).

**Table 1.** Examples of the current clinical uses of patient-reported outcomes in UK primary care^a^

Use	**Description**	**Example**
Assessing severity of symptoms	Highlight patients’ experiences of symptoms related to a health condition or treatment	Epworth Sleep Scale^67^
Informing treatment decisions	Inform patient—physician discussions and enable shared decision making	Patient Health Questionnaire –9 (PHQ-9)^68^
Tracking outcomes	Allows patients and providers to observe important trends, and adjust care accordingly	Asthma Quality of Life Questionnaire^69^
Facilitating provider patient discussions	Allow patients to provide information about their health, concerns, and priorities, and identify topics for discussion during the clinical visit	General Health Questionnaire (GHQ-12)^70^
Monitoring health and wellbeing	Routinely collected measures related to general health and wellbeing, provides important information about an individual’s overall health	International Prostate Symptom Score (IPSS)^71^

^a^after Lavallee *et al*,^5^ Turner *et al* 2019.^72^

Despite their widespread use, little is known of how successfully PROMs have been integrated into primary care or of the benefits that result.^16^ Here the findings are reported from a series of semi-structured interviews with GPs, which have been analysed using a framework-based approach. The approach permitted a comprehensive evaluation of the existing use of PROMs within five domains ranging from the characteristics of the individual PROM to the overarching processes and policies guiding their use.^17^ This helped to identify the key areas that need to be addressed if PROMs are to be successfully implemented as part of the long-term plan for the NHS.

## Method

### Design

The authors used a series of semi-structured interviews with GPs across England and a deductive content analysis^18^ to populate the domains of the CFIR.^19^ The CFIR consists of 39 constructs presented within five key domains: (1) intervention characteristics, relating to the design and development of the intervention; (2) outer setting, referring to the influence of the environment external to the organisation; (3) inner setting, describing factors integral to the organisation; (4) characteristics of individuals, consisting of the knowledge and beliefs of stakeholders; and (5) process of implementation that entails the planning and management of the implementation of an intervention. The CFIR has been successfully used in the post-hoc deductive analysis of qualitative data,^18^ and its conceptual clarity has enabled it to capture the complexity of implementation at multiple sites and in various settings.^14,17,20–22^

### Recruitment and consent

Participants were recruited in two ways. First, GPs were invited to take part in a survey of PROMs use through Doctors.net.uk, an online network of GPs^23^ (at the end of which, the 100 who took part were asked to indicate their willingness to participate in a semi-structured interview). Second, GPs were recruited through convenience sampling^24^ identified by members of the research team, one of whom was previously known by the interviewer IL. Verbal audio-recorded consent was taken prior to the start of each interview. The intention was to conduct a minimum of 25 interviews considered an appropriate sample size for a study of this type.^25^

### Data collection

All interviews were conducted one to one between June and September 2018. An interview schedule was used that included questions on which, if any, PROMs were used by each participant or members of their practice team, and why (see Box 1). They took place via telephone for the convenience of participants and were conducted by the first author (IL), a research fellow employed by the University of Birmingham, who is trained and experienced in qualitative research with an interest in health service delivery, but with no previous contact with any participant. They proceeded until 25 interviews had been conducted, by which point data saturation was reached as no new answers were being received.^26^ All interviews were digitally recorded and transcribed verbatim by a professional transcription company, and the data managed using NVivo (version 10).

Box 1 **Topic guide for semi-structured interviews**

**Use of PROMs in care**

- For which reasons do you currently use PROMs?

- Prompts: screening, diagnosis, monitoring, shared decision making (care plans, end-of-life care), Quality Outcomes Framework, other?

- What influences your choice on the PROMs you use?

- Prompts: how do you hear about the PROMs that are available? Is there a standard set of PROMs used across the practice? Who recommends them (clinical commissioning group, colleague, other)

- How frequently do you use PROMs?
- Which individuals use PROMs in your organisation?

- Prompts: clinical staff, for example, GPs, nurse practitioners, healthcare assistants, community matron, physician associate; non-clinical staff, for example, receptionists, administrators, practice managers; patient participation group (in partnership with practice)

- How do you use the data that are gained from PROMs?

- Prompts: how do they inform care? Evaluation of practice performance? Resource allocation? Who is involved and how?

**Future PROM use**

- Which areas or groups of patients (if any) do you think PROMs could be used more effectively?

- Prompts: multimorbidity, metric of care (patient perception), carers, part of discharge note? Other?

- What factors would facilitate the effective use of PROMs?

- Prompts: integration with existing systems or processes, integration with electronic health record, automatically generated and distributed to patients based on algorithm or diagnosis, utility and usability (practical or useful or relevant), recommended by trusted source

- What are the main barriers to increased use of PROMs?

### Analysis

The five domains and sub-constructs of the CFIR were used as a framework for a deductive content analysis, that is, one based on the prior understanding of the concept being analysed.^18^ The transcripts were analysed independently by IL and SG. In both instances the data were searched for text relating to the framework, and the issues that emerged within each of the five domains were discussed and agreed on by both.

## Results

Of the 25 GPs interviewed, 18 were recruited following the survey and a further seven from convenience sampling.^24^ They had a range of clinical experience and were employed at 25 practices across England, representing 21 different clinical commissioning groups (CCGs). Just over half were male,^14^ and the number of years qualified ranged from 2–33. The interviews lasted from between 18 and 59 minutes, with an average length of just under 29 minutes. Table 2 summarises participants’ characteristics and practice location by region.

**Table 2.** Summary characteristics of participants

**Sex** **,** ***n*** **(%** **)**									
Female					Male				
12 (48)					13 (52)				
**Years qualified** **,** ***n*** **(%** **)**									
1–5		6–10		11–15			16–20	>20	
4 (16)		5 (20)		9 (36)			3 (12)	4 (16)	
**Region** **,** ***n*** **(%** **)** ^a^									
North East	East Midlands		South East		South	West Midlands			North West
4 (16)	1 (4)		5 (20)		4 (16)	5 (20)			5 (20)

^a^One GP was a locum and could not be allocated to a region.

Within the five domains of the CFIR framework, each pre-existing construct that was shared by the data were populated with the issues that emerged from the discussions with GPs. Not all 39 constructs of the framework emerged from the data.^27^ The domain, existing construct (with definition), and emerging issues are summarised in Table 3 and a description of each domain, the relevant construct, and exemplar quotes for each theme are provided below.

**Table 3.** Summary of the issues identified in relation to the key domains of the CFIR, and its existing constructs

Domain	Existing CFIR construct^19^	Definition within CFIR^19^	Emergent issues
I. Intervention characteristics	*Evidence strength and quality*	The stakeholder’s belief in the quality and validity of the evidence of the intervention having the desired outcome	Evidence for the benefits of PROMs use in primary care
	*Relative advantage*	The perceived advantage of using a particular intervention versus an alternative or existing solution	PROMs may be used to frame discussions of shared-decision making, justify treatment decisions, support nursing staff
	*Adaptability*	The degree to which an intervention can be refined to meet the specific needs of the local environment	Ability to be digitalised
	*Complexity*	The potential disruptiveness and intricacy involved in its implementation	PROMs can take time to complete and to interpret and utilise results
	*Design quality*	How well the intervention is assembled and presented	PROMs are poorly presented to stakeholders
II. Outer setting	*Patient needs and resources*	The requirements of patients and the factors that influence how they are met by an organisation	Variation in patient reliability, health literacy, comorbidities
	*External policy and incentives*	The strategies that policymakers and commissioners employ to spread the implementation of the intervention, include mandates, guidelines, and financial incentives	Adverse influence of financial incentives, CCG
III. Inner setting	*Implementation climate*	The capacity for change of an organisation through its attitude to the intervention, their relative priority, and how their use will be supported and rewarded	No pressure for change, compatibility, relative priority
	*Readiness for implementation*	The tangible indicators of the decision to implement a particular intervention this includes factors such as access to knowledge, information, and training	GPs lack of awareness of PROMs, no systematic training
IV. Characteristics of individuals	*Knowledge and beliefs about the intervention*	The attitudes of individual GPs towards any intervention as shaped by their understanding of its use and the value they place on it	Considered a research tool
V. Process	*Planning*	The engagement of organisations and individuals in the process of change and ensuring an appropriate plan is in place	Lack of coherent approach
	*Engagement*	The degree to which relevant individuals are engaged in the process of implementation	Absence of consultation or staff engagement

CCG = clinical commissioning group. CFIR = Consolidated Framework of Implementation Research PROMs = Patient Reported Outcome Measures.

### Intervention characteristics

The participants raised issues relating to the evidence base for PROMs, their relative advantage, adaptability, and the overall quality of their design.

#### Evidence strength and quality

There is a perceived lack of evidence of the efficacy of PROMs in primary care whether for improving patient satisfaction or outcomes, or on wider service utilisation. For example, one GP described how they would prefer to see the evidence of the benefits of using PROMs:

*'* *If there were validated tools which were validated and evidence-based to show improved quality of care that would be helpful. Not only that but we were informed of what their value was and given some examples of how they worked* *… I think in modern medical culture, evidence-* *based medicine is very important, so if there’s something with evidence we can use it.* *'* (GP8 [study ID], male [sex], 28-years qualified [years since qualifying as a GP], South East [region])

#### Relative advantage

GPs described the advantages of using PROMs in comparison with standard care in a number of different areas; for example, PROMs helped provide a framework for shared decision making:

*'* *It does help direct the discussion regarding future management, especially the mental health patients because it allows them to objectively score how they feel and what’s going on, and allows me to help discuss treatment options with them.* *'* (GP12, male, 4-years qualified, West Midlands)

Another benefit of using PROMs as part of a consultation was their ability to provide quantitative evidence in support of a particular course of action:

*'* *I think they are quite useful nowadays when patients want reasons for things* *—* *I don’t blame patients for that at all, it’s perfectly reasonable* *—* *but you’ve got to be able to justify your actions* *…* *we should be able to justify our actions, and they are quite useful tools in that.* *'* (GP5, female, 20-years qualified, North East)

The use of PROMs is not confined solely to GPs and their value as prompts and support for other clinical staff on the practice team was also described:

*'* *Advanced nurse practitioners and practice nurses perhaps might be more inclined to use them, they might be a bit less confident about their underpinning medical knowledge. They work on commonality although we’ve got some excellent clinicians amongst them, they don’t have the rare and the unusual learning background, so they might be more likely to use them* *…* *'* (GP20, female, 31-years qualified, North West)

#### Adaptability

The group described how the majority of PROMs were completed as paper copies. One GP described how being able to capture patient responses electronically would enhance their usability:

*'* *I think they clearly need to be captured in a coded way; they need to be capturable, potentially independent of the consultation. So I don’t think a PROM captured as a result of* *“* *now I’ve done your diabetic check, are you satisfied with it?”, is necessarily a valid or appropriate way of doing it. I know that there are quite a lot of systems used for texting patients to remind them of their appointments or to ask them to book appointments, and I would think building the PROMs into those platforms so it’s automated and not time consuming to collect them is probably the best way to go.* *'* (GP1, female, 29-years qualified, South East)

#### Complexity

A number of participants felt that the time it takes to complete, analyse, and usefully integrate the additional sources of information provided by PROMs could impact on their use:

*'* *Yeah, in a pressurised rushing surgery and you’ve only got ten minutes the person usually would need at least* *20* *minutes* *to solve their issues, and if you were to include a questionnaire on top of that you would be definitely talking about* *30* *minutes* *at least, and you can’t afford to be doing that on a regular basis. You can do it as a one* *-* *off thing and then you have an idea, but you would be pressurised to just do things quickly* *...* *'* (GP2, male, 10-years qualified, North West)

#### Design quality

The lack of an engaging narrative from policymakers or commissioners as to why PROMs were appropriate was described. One GP noted how their branding or presentation could be improved to positively influence attitudes to their uptake:

*'* *…* *it’s putting it in a lively interesting way that isn’t telling me* *“* *this is something I have to do as part of my job* *”* , and it’s not yet another mandatory training that’s just sh*t awful that I have to sit through for *3* *hours every* *3* *years* *… So making it so that it’s something that people might want to look at,* *“* *Look at this! This is interesting!* *'* (GP20, female, 31-years qualified, North West)

### Outer setting

Participants described the impact of patients and their various needs and resources, and the influence of external policy and incentives.

#### Patient needs and resources

Dependent on location and the demographic of the local population, the ability of patients to understand the concept of a PROM or its constituent items varied. One GP described the difficulties of using PROMs in populations with poor levels of literacy:

*'* *I do work in a slightly deprived population so we do come across some patients who can’t read or write, or who may not have a good understanding of English, so may not fully understand the questions that are being asked of them.*' (GP12, male, 4-years qualified, West Midlands)

Concern was expressed about how the reliability of a PROM might be undermined when completed by patients willing to manipulate the output to serve their own ends:

*'* *I think it would be a waste of time to give a hypochondriac frequent attender a questionnaire about all of their conditions, because then you have to document* *—* *sorry for being so honest* *—* *but if you have to document your hypochondriac scores for the marks on depression … because people are capable of exaggerating on questionnaires, and if there is the type that is a healthy predisposition to do so then I think that’s another can of worms for us.* *'* (GP13, male, 10-years qualified, South East)

Another reason why it was felt patients might filter their responses was to provide the answers they believe the clinician would prefer:

*'* *…* *sometimes the patient can fill them in with what they think the clinician might want them to say rather than what they actually feel. So sometimes patients can underplay their symptoms, and equally sometimes patients can overplay their symptoms if there might be some perhaps secondary gain for them in terms of certification from work or whether they want some help with some other part of their care. So I think they can potentially be a bit skewed by that.* *'* (GP19, male, 8-years qualified, West Midlands)

A growing number of patients have multimorbidities,^28^ and some GPs voiced concerns that using a PROM directed toward a single condition would not produce a reliable result for these patients:

*'* *I think there’s that whole issue of multi* *morbidity, and not actually capturing what you want to capture because it’s so hard to tease out what that issue of diagnostic overshadowing what condition is causing what symptom.* *'* (GP24, female, 9-years qualified, East Anglia)

#### External policy and incentives

The influence of the Quality Outcomes Framework (QOF)^29^ on the decision of some GPs to use the PROM Patient Health Questionnaire-9 (PHQ-9) was described:

*'* *So the big one is PHQ-9, it’s pushed very hard and for example with people with chronic diseases as well it flags up in the QOF box on EMIS* [clinical management system]*. But in reality it’s irrelevant to assisting you that much in terms of referral and management, so there’s no point in doing it.* *'* (GP18, male, 2-years qualified, West Midlands)

### Inner setting

Participants described the influence of the organisational environment, and the readiness of that organisation for change.

#### Implementation climate

There appeared little impetus for changing existing ways of working to incorporate PROMs and their training meant they could gather the same information without using PROMs, which could actually impede patient-focused conversations:

*'* *I think there’s a role and a value to having a PROM but I don’t think it replaces a face* *-* *to* *-* *face discussion with patients.* *'* (GP12, male, 4-years qualified, West Midlands)

*'* *Perhaps it a gap in my practice, I don’t know, but personally I find that seeing a patient with as few distractions as possible to get* *… sometimes I ask them to make a diary of their symptoms or something like that but that would be their own interpretation of what I have asked them to do rather than somebody else’s interpretation of what they be expected to feel, or might put ideas into their head a little bit.* *'* (GP19, male, 8-years qualified, West Midlands)

The implementation of PROMs into everyday practice seemed a relatively low priority. One GP noted that current processes already produce an abundance of patient data and there was little incentive to collect more:

*'* *…* *when you talk about the frailer ones, the ones who have all the diseases, all the medicines, and they’re common as well, they are not* *… your priority with them is not filling out a PROM, it’s about trying to actually get them functionally better, and trying to* *… and not be bamboozling them with lots of extra questions. It’s examining them and looking at what you actually need to do to improve their care rather than just trying to capture how bad they are right now* *…* *'* (GP24, female, 9-years qualified, East Anglia)

#### Readiness for implementation

Some of those spoken to described how they were unaware of which PROMS were available even within a single condition:

*'* *I think that forever more are appearing, COPD* [chronic obstructive pulmonary disease] *have got a whole range of them now as well, about patient’s feeling of breathlessness and stuff. I think the difficulty is remembering which disease now has one … I have never been trained; perhaps I am doing it wrong. I stick the piece of paper in front of the patient and they look at it and ask them if they need any help. That’s probably a terrible way of doing it, I don’t know. If we need training I don’t know when we’re going to get it, but probably I am terrible at it, I don’t know.* *'* (GP5 female, 20-years qualified, North East)

### Characteristics of individuals

The attitude of GPs to the use of PROMs would vary in line with their personal knowledge and beliefs.

#### Knowledge and beliefs

Differences between providers were noted as to the perceived role of PROMs with some describing their worth only as a research tool:

*'* *I think PROMs tend to* *… the thing with a lot of PROMs they tend to be very useful in research, and not so useful in actual daily practice, and that’s where* *… if you’re going to use a PROM it needs to streamline your service not add more time to it.* *'* (GP18, male 2-years qualified, West Midlands)

Others felt that PROMs had the potential to make a positive contribution to patient experience and outcome if used correctly:

*'* *Ultimately I think they could be really useful in many situations but sadly I think the way they are used at the moment probably doesn’t maximise their benefit and actually they are probably seen as more of a nuisance than a value, really, in most ways that they are used currently. So yeah I think there’s work to be done.* *'* (GP23, female, 8-years qualified, North East)

### Process

The GPs interviewed described issues with both the strategic planning for the use of PROMs and the engagement of staff in the process.

#### Planning

Participants described how the introduction of PROMs into their practice occurred on an ad hoc basis. For example, after they have learnt about them at a particular course they attended:

*'* *I tend to find if I’ve been on a course and they will tell me about a PROM I will use it for a few days and then I’ll forget about it, but that’s probably what I’ll tend to do, if I’m on a course and they just share a PDF of it I’ll give it a try and see if I like it.*' (GP15, male, 17 years qualified, North West)

Another described how their use of a PROM would be inspired by multiple recommendations from a variety of uncoordinated sources:

*'* *… I am unlikely to go and start using some new coeliac disease PROM when I have just been to a talk from a private gastroenterologist or something like that. I am more likely to use something that is appearing to me in lots of different areas of my CPD* [continuing professional development] *or medical education. So I might see a paper about it, and then I might hear a colleague talking about it, and then I might see something on GP Notebook or something like that. So you’re getting over exposed to it, and then try it out and see how well it resonates, and how useful it is and how quick and easy to remember it is.* *'* (GP24, female, 9-years qualified, East Anglia)

#### Engagement

The degree to which relevant individuals are engaged in the process of implementation can affect its success.^19^ For example, GPs were resistant to the use of PROMs when introduced as a mandatory aspect of the referral pathway:

*'* *I think a lot of GP colleagues my feeling is that they don’t like anything compulsory, so when the CCG said “you need to fill in this score otherwise we will reject the referral” I’ll tell you that didn’t go down very well with everyone.* *'* (GP13, male, 10-years qualified, South East)

Another GP noted that robust evidence of their efficacy was more persuasive than any subjective recommendation from a CCG with contrasting priorities to the clinicians:

*'* *If the CCG say we had to use it* *… then we would use it, but just because the CCG says doesn’t put* [my] *trust in it really because they do it for political reasons and bureaucratic reasons, not necessarily medical reasons. For me I am quite evidence-based, personally, and if someone was to show me* *… I’m the outlier and most GPs love PROMs and I would actually be thinking* *"* *hang on I’m the outlier here, actually maybe I’ll just get more on board* *"* *. If there was a study saying this particular PROM if they said PHQ-9 shortened a ten minute consultation down to five minutes, improves on patient outcomes, reduces re-attendance rates, improve compliance to medications, then I would say “right we’ve got to get on board and do that”.* *'* (GP18, male, 2-years qualified, West Midlands)

## Discussion

### Summary

GPs described uncertainty about the efficacy of PROMs and whether they offer any advantage over more traditional patient consultations. Concerns were also voiced about the reliability of a single tool when used on patients with varied needs, abilities, and comorbidities. Some felt that although PROMs had the potential to be digitally integrated into existing systems, they were currently poorly packaged and presented. There appeared to be little offered to GPs in the way of guidance, whether from their practice, commissioning group, or policymakers. This was reflected in the apparent absence of training, and the lack of staff engagement or awareness of any coordinated implementation strategy.

### Strengths and limitations

This is the first time GP perspectives have been sought on the current use of PROMs in English primary care. Interviews were conducted across England with a balance of sexes and a range of experience. GPs recruited via both sampling methods shared similar views. There is the potential of bias from the self-selection of the participants;^30^ however, a range of views were captured and the findings reflected previous work that described diverse levels of adoption and utilisation in primary care.^16^

### Comparison with existing literature

#### Intervention characteristics

The minimal design of PROMs enables their integration with existing IT platforms and has contributed to the political enthusiasm for the expansion of their use in clinical practice.^31^ Multiple digital platforms can enable the independent completion of PROMs by patients, and produces readily interpretable outputs for patients and clinicians.^32–35^

There is a lack of coherent and clearly communicated evidence of the benefit of using PROMs in routine primary care.^36^ One frequently reported advantage in discrete settings, such as oncology^37,38^ and psychiatry,^39,40^ is their aid to clinician—patient communication,^37,38,41,42^ contributing to improved quality and experience of care.^41,43–49^ However, this is not necessarily applicable to the broader extent of primary care.^14,50,51^ Extending PROMs to account for this complexity runs counter to existing evidence indicating that clinicians prefer PROMs with fewer items^52–55^ and simplicity of scoring.^56^

#### Outer setting

PROMs have the ability to encourage patient engagement,^36,50,57^ and patients in acute settings considered them a valuable opportunity to receive clinical guidance or feedback.^57^ This is in contrast to busy clinicians, for whom PROMs exemplify the excess of data they are expected to review.^57^ These concerns over additional workload were compounded for some of the participants by doubts in the veracity of data provided by patients with a range of cognitive abilities, health literacy, and motivations.

#### Inner setting

A lack of awareness among GPs was found as to which PROMs were available and they had received little training in their use. In other settings the uptake of PROMs has been limited by a lack of staff engagement in their deployment,^50,58–60^ including the content and delivery of related training.^59,61^

#### Characteristics of individuals

The ways in which PROMs might be incorporated into routine care is shaped by clinician beliefs about their roles and responsibilities.^50^ GPs are trained to solve problems independently^62^ and there were concerns using PROMs might diminish independent clinical thought. Being sympathetic to clinician autonomy might overcome such misgivings^63^ and encourage their uptake into routine care.^58–61^

#### Process

A whole-systems approach that flows from policymakers to individual providers has been recommended for implementing PROMs in primary care.^32,36,58^ However, a coherent national strategy for PROM implementation is yet to emerge.^6,64^ In the UK, financial incentives have previously been used to encourage the uptake of PROMs in primary care such as the use of PHQ-9 as part of QOF.^29^ Such isolated monetary drivers are, however, renowned for attempts to ‘game’ the system and encourage inappropriate use.^61,65^

### Implications for research and practice

In the US, a coherent strategy has been developed for implementing PROMs across a range of conditions and healthcare settings,^35^ and a number of characteristics shared by successful implementation programmes have emerged.^66^ These include robust IT systems, integration into clinical pathways, and training of patients and professionals in their use.^8^ The primary objective in the UK must be to create and communicate a compelling body of evidence of their efficacy and benefits. This should include identification of which PROM is appropriate in which circumstance, engaging staff throughout the process. If PROMs are to make a contribution to the long-term future of the NHS, policymakers and commissioners need to construct a coherent implementation strategy, which is appropriately planned, funded, and adapted following rigorous and ongoing evaluation.

## References

[1] NHS England (2019). The NHS long term plan. https://www.longtermplan.nhs.uk/wp-content/uploads/2019/01/nhs-long-term-plan-june-2019.pdf.

[2] Royal College of General Practitioners (2019). Personalised care. https://www.rcgp.org.uk/clinical-and-research/our-programmes/about-person-centred-care.aspx.

[3] NHS England (2019). Personalised care. https://www.england.nhs.uk/personalisedcare/.

[4] NHS England (2019). Comprehensive model of personalised care. https://www.england.nhs.uk/personalisedcare/comprehensive-model-of-personalised-care/.

[5] Lavallee DC, Chenok KE, Love RM (2016). Incorporating patient-reported outcomes into health care to engage patients and enhance care. Health Aff.

[6] Calvert M, Kyte D, Price G (2019). Maximising the impact of patient reported outcome assessment for patients and society. BMJ.

[7] Basch E, Snyder C (2017). Overcoming barriers to integrating patient-reported outcomes in clinical practice and electronic health records. Ann Oncol.

[8] Basch E, Barbera L, Kerrigan CL (2018). Implementation of patient-reported outcomes in routine medical care. Am Soc Clin Oncol Educ Book.

[9] Department of Health (2007). Department of Health annual report 2006 to 2007.

[10] Department of Health (2008). Department of Health annual report 2007 to 2008.

[11] Arpinelli F, Bamfi F (2006). The FDA guidance for industry on pros: the point of view of a pharmaceutical company. Health Qual Life Outcomes.

[12] U.S. Department of Health and Human Services FDA Center for Drug Evaluation and Research, U.S. Department of Health and Human Services FDA Center for Biologics Evaluation and Research, U.S. Department of Health and Human Services FDA Center for Devices and Radiological Health (2006). Guidance for industry: patient-reported outcome measures: use in medical product development to support labeling claims: draft guidance. Health Qual Life Outcomes.

[13] Greenhalgh J, Dalkin S, Gooding K (2017). Functionality and feedback: a realist synthesis of the collation, interpretation and utilisation of patient-reported outcome measures data to improve patient care.

[14] Foster A, Croot L, Brazier J (2018). The facilitators and barriers to implementing patient reported outcome measures in organisations delivering health related services: a systematic review of reviews. J Patient Rep Outcomes.

[15] Hjollund NHI (2017). Individual prognosis of symptom burden and functioning in chronic diseases: a generic method based on patient-reported outcome (pro) measures. J Med Internet Res.

[16] Peters M, Crocker H, Jenkinson C (2014). The routine collection of patient-reported outcome measures (PROMs) for long-term conditions in primary care: a cohort survey. BMJ Open.

[17] Kirk MA, Kelley C, Yankey N (2015). A systematic review of the use of the consolidated framework for implementation research. Implementation Sci.

[18] Ilott I, Gerrish K, Booth A (2013). Testing the consolidated framework for implementation research on health care innovations from South Yorkshire. J Eval Clin Pract.

[19] Damschroder LJ, Aron DC, Keith RE (2009). Fostering implementation of health services research findings into practice: a consolidated framework for advancing implementation science. Implement Sci.

[20] Acosta J, Chinman M, Ebener P (2013). An intervention to improve program implementation: findings from a two-year cluster randomized trial of Assets-Getting to Outcomes. Implement Sci.

[21] Cragun D, DeBate RD, Vadaparampil ST (2014). Comparing universal Lynch syndrome tumor-screening programs to evaluate associations between implementation strategies and patient follow-through. Genet Med.

[22] English M, Nzinga J, Mbindyo P (2011). Explaining the effects of a multifaceted intervention to improve inpatient care in rural Kenyan hospitals — interpretation based on retrospective examination of data from participant observation, quantitative and qualitative studies. Implement Sci.

[23] Doctors.net.uk (2020). The UK's largest professional network of 240,239 doctors. https://www.doctors.net.uk/.

[24] Suen L-JW, Huang H-M, Lee H-H (2014). [A comparison of convenience sampling and purposive sampling]. Hu Li Za Zhi.

[25] Sandelowski M (1995). Sample size in qualitative research. Res Nurs Health.

[26] Guest G, Bunce A, Johnson L (2006). How many interviews are enough? An experiment with data saturation and variability. Field Methods.

[27] Keith RE, Crosson JC, O'Malley AS (2017). Using the consolidated framework for implementation research (CFIR) to produce actionable findings: a rapid-cycle evaluation approach to improving implementation. Implement Sci.

[28] Kingston A, Robinson L, Booth H (2018). Projections of multi-morbidity in the older population in England to 2035: estimates from the population ageing and care simulation (PACSim) model. Age Ageing.

[29] NHS Digital (2020). Quality and Outcomes Framework (QOF), enhanced services and core contract extraction specifications (business rules). https://digital.nhs.uk/data-and-information/data-collections-and-data-sets/data-collections/quality-and-outcomes-framework-qof.

[30] Marshall MN (1996). Sampling for qualitative research. Fam Pract.

[31] Lavallee DC, Austin E, Franklin PD (2018). How can health systems advance patient-reported outcome measurement?. Jt Comm J Qual Patient Saf.

[32] Bantug ET, Coles T, Smith KC (2016). Graphical displays of patient-reported outcomes (pro) for use in clinical practice: what makes a pro picture worth a thousand words?. Patient Educ Couns.

[33] Gibbons C, Bower P, Lovell K (2016). Electronic quality of life assessment using computer-adaptive testing. J Med Internet Res.

[34] Graham AK, Minc A, Staab E (2019). Validation of the computerized adaptive test for mental health in primary care. Ann Fam Med.

[35] Broderick J, DeWit EM, Rothrock N (2013). Advances in patient reported outcomes: the NIH PROMIS measures. EGEMS.

[36] Wheat H, Horrell J, Valderas JM (2018). Can practitioners use patient reported measures to enhance person centred coordinated care in practice? A qualitative study. Health Qual Life Outcomes.

[37] Howell D, Molloy S, Wilkinson K (2015). Patient-Reported outcomes in routine cancer clinical practice: a scoping review of use, impact on health outcomes, and implementation factors. Ann Oncol.

[38] Kotronoulas G, Kearney N, Maguire R (2014). What is the value of the routine use of patient-reported outcome measures toward improvement of patient outcomes, processes of care, and health service outcomes in cancer care? A systematic review of controlled trials. J Clin Oncol.

[39] Knaup C, Koesters M, Schoefer D (2009). Effect of feedback of treatment outcome in specialist mental healthcare: meta-analysis. Br J Psychiatry.

[40] Krägeloh CU, Czuba KJ, Billington DR (2015). Using feedback from patient-reported outcome measures in mental health services: a scoping study and typology. Psychiatr Serv.

[41] Luckett T, Butow PN, King MT (2009). Improving patient outcomes through the routine use of patient-reported data in cancer clinics: future directions. Psychooncology.

[42] Alsaleh K (2013). Routine administration of standardized questionnaires that assess aspects of patients' quality of life in medical oncology clinics: a systematic review. J Egypt Natl Canc Inst.

[43] Chen J, Ou L, Hollis SJ (2013). A systematic review of the impact of routine collection of patient reported outcome measures on patients, providers and health organisations in an oncologic setting. BMC Health Serv Res.

[44] Etkind SN, Daveson BA, Kwok W (2015). Capture, transfer, and feedback of patient-centered outcomes data in palliative care populations: does it make a difference? A systematic review. J Pain Symptom Manage.

[45] Marshall S, Haywood K, Fitzpatrick R (2006). Impact of patient-reported outcome measures on routine practice: a structured review. J Eval Clin Pract.

[46] Espallargues M, Valderas JM, Alonso J (2000). Provision of feedback on perceived health status to health care professionals: a systematic review of its impact. Med Care.

[47] Santana M-J, Feeny D (2014). Framework to assess the effects of using patient-reported outcome measures in chronic care management. Qual Life Res.

[48] Wasson JH, Stukel TA, Weiss JE (1999). A randomized trial of the use of patient self-assessment data to improve community practices. Eff Clin Pract.

[49] Valderas JM, Alonso J (2008). Patient reported outcome measures: a model-based classification system for research and clinical practice. Qual Life Res.

[50] Greenhalgh J, Dalkin S, Gibbons E (2018). How do aggregated patient-reported outcome measures data stimulate health care improvement? A realist synthesis. J Health Serv Res Policy.

[51] Valderas JM, Kotzeva A, Espallargues M (2008). The impact of measuring patient-reported outcomes in clinical practice: a systematic review of the literature. Qual Life Res.

[52] Mitchell AJ, Coyne JC (2007). Do ultra-short screening instruments accurately detect depression in primary care? A pooled analysis and meta-analysis of 22 studies. Br J Gen Pract.

[53] Haywood KL, Staniszewska S, Chapman S (2012). Quality and acceptability of patient-reported outcome measures used in chronic fatigue syndrome/myalgic encephalomyelitis (CFS/ME): a systematic review. Qual Life Res.

[54] Krebs EE, Bair MJ, Carey TS (2010). Documentation of pain care processes does not accurately reflect pain management delivered in primary care. J Gen Intern Med.

[55] Löwe B, Kroenke K, Gräfe K (2005). Detecting and monitoring depression with a two-item questionnaire (PHQ-2). J Psychosom Res.

[56] Kroenke K (2012). Enhancing the clinical utility of depression screening. CMAJ.

[57] Reading MJ, Merrill JA (2018). Converging and diverging needs between patients and providers who are collecting and using patient-generated health data: an integrative review. J Am Med Inform Assoc.

[58] Antunes B, Harding R, Higginson IJ, EUROIMPACT (2014). Implementing patient-reported outcome measures in palliative care clinical practice: a systematic review of facilitators and barriers. Palliat Med.

[59] Boyce MB, Browne JP, Greenhalgh J (2014). The experiences of professionals with using information from patient-reported outcome measures to improve the quality of healthcare: a systematic review of qualitative research. BMJ Qual Saf.

[60] Duncan EAS, Murray J (2012). The barriers and facilitators to routine outcome measurement by allied health professionals in practice: a systematic review. BMC Health Serv Res.

[61] Greenhalgh J, Dalkin SM, Gibbons E (2017). How does PROMs feedback work as a quality improvement strategy? Lessons from the feedback of other forms of performance data. Qual Life Res.

[62] Royal College of General Practitioners Training and practice. https://www.rcgp.org.uk/training-exams/.

[63] Rotenstein LS, Huckman RS, Wagle NW, Patients M (2017). Making patients and doctors happier — the potential of patient-reported outcomes. N Engl J Med.

[64] Kyte D, Cockwell P, Lencioni M (2016). Reflections on the national patient-reported outcome measures (PROMs) programme: where do we go from here?. J R Soc Med.

[65] Year of Care Partnerships (2011). Year of Care. Report of findings from the pilot programme. https://www.yearofcare.co.uk/sites/default/files/images/YOC_Report.pdf.

[66] Evans JP, Smith A, Gibbons C (2018). The National Institutes of health patient-reported outcomes measurement information system (PROMIS): a view from the UK. Patient Relat Outcome Meas.

[67] The Epworth Sleepiness Scale (1988). About the ESS. https://epworthsleepinessscale.com/about-the-ess/.

[68] Kroenke K, Spitzer RL, Williams JB (2001). The PHQ-9: validity of a brief depression severity measure. J Gen Intern Med.

[69] Qoltech Measurement of health-related quality of life & asthma control. https://www.qoltech.co.uk/aqlq.html.

[70] GL Assessment (2017). General health questionnaire: identify minor psychiatric disorders. https://www.gl-assessment.co.uk/products/general-health-questionnaire-ghq/.

[71] Urological Sciences Research Foundation (USRF) International prostate symptom score (IPSS). http://www.usrf.org/questionnaires/AUA_SymptomScore.html.

[72] Turner GM, Litchfield I, Finnikin S (2020). General practitioners' views on use of patient reported outcome measures in primary care: a cross-sectional survey and qualitative study. BMC Fam Pract.

